# XIAP’s Profile in Human Cancer

**DOI:** 10.3390/biom10111493

**Published:** 2020-10-29

**Authors:** Huailu Tu, Max Costa

**Affiliations:** Department of Environmental Medicine, Grossman School of Medicine, New York University, New York, NY 10010, USA; ht1204@nyu.edu

**Keywords:** XIAP, apoptosis, cancer, therapeutics, non-coding RNA

## Abstract

XIAP, the X-linked inhibitor of apoptosis protein, regulates cell death signaling pathways through binding and inhibiting caspases. Mounting experimental research associated with XIAP has shown it to be a master regulator of cell death not only in apoptosis, but also in autophagy and necroptosis. As a vital decider on cell survival, XIAP is involved in the regulation of cancer initiation, promotion and progression. XIAP up-regulation occurs in many human diseases, resulting in a series of undesired effects such as raising the cellular tolerance to genetic lesions, inflammation and cytotoxicity. Hence, anti-tumor drugs targeting XIAP have become an important focus for cancer therapy research. RNA–XIAP interaction is a focus, which has enriched the general profile of XIAP regulation in human cancer. In this review, the basic functions of XIAP, its regulatory role in cancer, anti-XIAP drugs and recent findings about RNA–XIAP interactions are discussed.

## 1. Introduction

X-linked inhibitor of apoptosis protein (XIAP), also known as inhibitor of apoptosis protein 3 (IAP3), baculoviral IAP repeat-containing protein 4 (BIRC4), and human IAPs like protein (hILP), belongs to IAP family which was discovered in insect baculovirus [1]. Eight different IAPs have been isolated from human tissues: NAIP (BIRC1), BIRC2 (cIAP1), BIRC3 (cIAP2), XIAP (BIRC4), BIRC5 (survivin), BIRC6 (apollon), BIRC7 (livin) and BIRC8 [2]. Like other human IAPs, XIAP inhibits cell death mainly through blocking apoptosis. However, unlike other human IAPs, XIAP is not very conserved in sequence and structure, hence it is also called human IAP like protein (hILP) [3].

XIAP was identified by the polymerase chain reaction (PCR) method using homologous IAP sequences from the X chromosome of *Cydia pomonella*. Therefore, it was named X-linked inhibitor of apoptosis protein. Further research revealed that XIAP is coded for by the Xiap gene located at Xq24-25 and this gene was present in numerous adult and fetal tissues, such as heart, brain, lung, and liver, but was undetectable in peripheral blood leukocytes. The transcript size of the Xiap gene is about 9.0 kb and contains a 1.8 kb open reading frame [4].

In addition to three IAPs characteristic BIR (baculoviral IAP repeat) motifs at the N-terminal region, full-length XIAP contains an UBA (ubiquitin-associated) domain followed by a RING finger domain. BIR domain contains a three-stranded antiparallel β-sheet and four α-helices [5]. Between the BIR1 and BIR2 domain, there is a special linker region which is critical in caspase interactions. Further NMR study revealed that the linker is the only contact site when XIAP interacts with caspase-7. The linker encompasses a major energetic determinant, while the BIR2 domain only serves a regulatory function during caspase binding and SMAC neutralization [6]. The RING domain primarily exhibits homodimer E3 ligase activity, mediating ubiquitination and degradation of many cellular proteins including XIAP itself [7]. Removing the RING domain stabilized XIAP in mouse thymocytes. However, instead of attenuating apoptosis, XIAP^ΔRING^ increases caspase activity and apoptosis, indicating that the RING domain is also essential in anti-apoptosis [8]. The UBA domain consists of triple α-helices with an additional N-terminal 3_10_ helix, which is mainly responsible for binding with some mono-ubiquitin and diubiquitins. The observation of XIAP–UBA dimerization in NMR spectroscopy provides another possible way for XIAP to self-associate [9].

**Figure 1:**
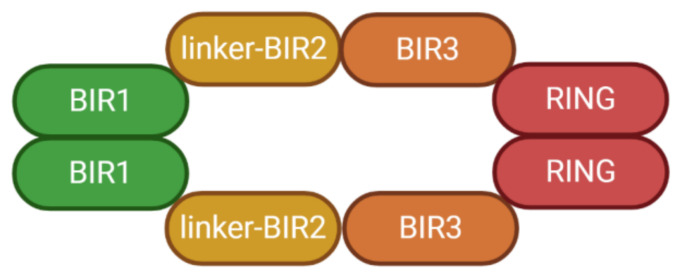
The structure of X-linked inhibitor of apoptosis protein (XIAP) homodimer. BIR: baculoviral IAP repeat. RING: RING (Really Interesting New Gene) finger doman.

SEC-MALS experiments indicated XIAP to be monodisperse in solution, forming an approximately 115KDa homodimer with its BIR1–BIR1 and RING–RING domains adhered together at the interface. The dimerization of XIAP monomers decide its quaternary structure, as shown in Figure 1. ICP-AES revealed 10 zinc ions per homodimer: one in each BIR domain and two for every RING domain. Preliminary studies of the full-length XIAP structure, demonstrated that the XIAP homodimer exhibited compact and rigid conformation (Figure 1) [10]. These findings have consequences when studying XIAP’s interaction with other molecules, such as BIR1–TAB1 and linker/BIR2–caspase3/7 interactions [11,12]. Polykertis et al. showed that XIAP must undergo conformational rearrangement before it can directly bind with other molecules [10]. However, the components involved, and the mechanisms are not well understood. Additional high-resolution studies on XIAP’s full-length structure and conformational transition from monodisperse state to binding state are needed. Representative models of the XIAP homodimer have identified the BIR1 and RING as two dimeric binding sites, however, the UBA domain is also relevant to XIAP self-association (vide supra). Whether the UBA domain is also a part of XIAP homodimer interface is not known.

## 2. XIAP and Cellular Functions

Apoptosis, a highly controlled process regulating cell death, is active from embryonic development until death. XIAP prevents cell apoptosis primarily by blocking the activation and maturation of caspase-3/-7/-9, which are major initiators and effectors of apoptosis [13,14]. There are several binding scenarios for XIAP and caspase interactions. The first involves the linker-BIR2 that binds to caspase-3/-7. Additionally, BIR3 has been reported to have the capability of binding and locking caspase-9 in an inactive monomeric state [15]. The inhibition of apoptosis by XIAP was thought to be independent of the RING domain since research revealed that XIAP can still inhibit apoptosis with a mutated or deleted RING domain [3], whereas later scientists found that XIAP^ΔRING^ increased apoptosis in mouse thymocytes [8]. This contradiction makes the RING domain function even more mysterious. From most of the experiments, we tend to accept that the BIR2/3 domains are the major components responsible for the anti-apoptosis activity of XIAP. Unlike BIR2/3, the BIR1 domain of XIAP does not possess the specific IBM-binding groove. Crystal structure revealed BIR1 to be another dimerization site of XIAP [16]. Butterfly-shaped BIR1/TAB1 dimer and BIR1 dimerization were indicated to be a critical step in XIAP-induced NF-κB activation [11]. Disruption of BIR1 dimerization using a BIR1 mutant XIAP^V80D^ and UBA domain mutant XIAP^ΔUBA^ abolished NF-κB activation. Since the XIAP UBA domain substitute, XIAP^cbl-b-UBA^, functions better in activating NF-κB, demonstrating that XIAP-induced NF-κB activation also resides in the UBA domain and its Ub-binding ability [17]. In addition to binding themselves or caspases, increasing studies also identified some possible binding substrates for each domain of XIAP as summarized in Table 1, which enriched XIAP’s functionality step by step.

**Figure 2:**
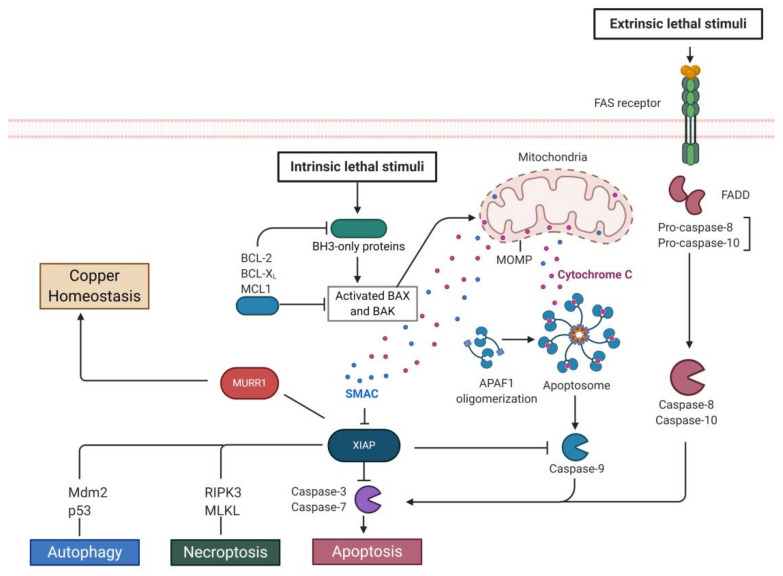
XIAP and cellular function. XIAP mainly inhibits caspases-3/7/9 through binding, which blocks the pre-apoptotic pathways. SMAC, a mitochondrial protein, blocks XIAP’s cellular functions by occupying its binding sites. XIAP switches to necroptosis by RIPK3 and mixed lineage kinase domain like pseudokinase (MLKL), mediates autophagy by Mdm2 and p53 and regulates copper homeostasis by MURR1.

Although, the main function of XIAP is to prevent apoptosis, additional roles are being discovered. Diablo homolog (DIABLO), also known as SMAC, is a mitochondrial inter-membrane space protein. SMAC–XIAP interaction prevents the XIAP binding to caspases and promotes cell apoptosis (Figure 2) [24]. Apart from apoptosis, XIAP also participates in the regulation of necroptosis, a cellular self-destruction program. The switch to necroptosis involves RIPK3 (Receptor-Interacting Serine/Threonine-Protein Kinase 3) and MLKL (Mixed Lineage Kinase domain Like pseudokinase), which were studied in mouse neutrophils [25]. XIAP is an autophagic modulator. Clinical studies show that the expression of XIAP is inversely associated with autophagy biomarker LC3 in hepatocellular carcinoma tissues [26]. At the same time, Huang et al. reported that XIAP inhibited autophagy and promoted tumorigenesis through the Mdm2-p53 mediated signaling pathway [27]. Additionally, XIAP limits caspase-8 dependent IL-1β processing, and also exhibits a regulatory role in innate immunity through NOD2 (Nucleotide-binding Oligomerization Domain-containing protein 2) signaling. Loss of XIAP is caused immunodeficiency and inflammation [28]. XIAP was also identified as a metalloprotein, since both of the three BIR domains and the RING domain possess zinc binding motifs [29]. Additionally XIAP is also a copper binding protein through all three BIR domains [29,30]. It has been established that XIAP is involved in maintaining copper homeostasis through negatively regulating MURR1, a recently found copper homeostasis factor. XIAP binds to and ubiquitinates MURR1 leading to its degradation without affecting XIAP’s anti-apoptotic property [31].

To date, XIAP is believed to be the most potent anti-apoptotic and death-preventing protein in existence. As displayed in Figure 2, XIAP is mainly responsible for anti-apoptosis, while it is also involved in autophagy, necroptosis and homeostasis maintenance. Dysregulation or inhibition of apoptosis is directly associated with cancer, autoimmune diseases and neurodegenerative disorders. Thus, abnormal expression of XIAP is a useful bio-marker for the early detection of cancer and other diseases (vide supra).

## 3. XIAP and Cancer

**Figure 3:**
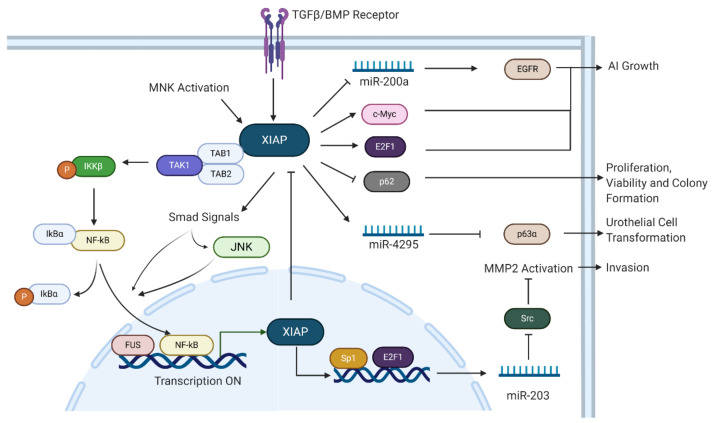
XIAP Signaling Pathways in Human Cancer. XIAP senses external signals through TGFβ and bone morphogenetic proteins (BMP) receptors. MNK activation and RPS3 (ribosomal protein S3) increase XIAP expression level. XIAP binds with TAB1, forming a XIAP-TAB1-TAB2-TAK1 complex, which further activates NF-κB and NF-κB-related downstream pathways. XIAP activates Smad signals which subsequently increases NF-κB and JNK activation, JNK activation alone can also switch on NF-κB pathway. Activated NF-κB and the FUS protein translocate into the nucleus, binding and initiating transcription of XIAP. Intranuclear XIAP binds to transcription factor Sp1 and E2F1, subsequently increasing transcription of miR-203 which further leads to MMP2 activation and invasion by inhibiting Src. Inhibition of miR-200a, stabilizing c-Myc and interacting with E2F1, XIAP facilitates anchorage-independent (AI) growth. XIAP promotes cell proliferation, viability and colony formation by blocking p62. Additionally, XIAP contributes to urothelial cell transformation through impairing p63α mRNA stability via miR-4295.

Apoptosis, necroptosis, autophagy and the immune response are all critical biochemical activities for homeostasis, and XIAP’s ability to impact these processes is indicative of its importance in the cell. Expression of human IAPs was analyzed in the National Cancer Institute panel of 60 human tumor cell lines, and XIAP was found to be expressed in a majority of these cell lines [32]. Dysregulation of XIAP has also been shown to impact the progression of multiple cancers. XIAP is involved in activation of TGFβ transcriptional mediators, NF-κB and JNK signaling pathways in a Smad4-dependent manner (Figure 3) [21]. XIAP enables cells to undergo malignant transformation, thus preventing cell death and initiating carcinogenesis. Apart from how XIAP contributes to cancer development, the mechanisms behind XIAP stabilization and up-regulation needs to be understood.

### 3.1. Bladder Cancer

Early diagnosis of bladder carcinoma remains a challenge. The combination of XIAP mRNA detection and voided urinary cytology achieved better specificity and sensitivity, demonstrating that XIAP was a non-invasive diagnostic biomarker in low-grade bladder cancer [33].

Both the BIR and RING domains of XIAP can promote anchorage-independent growth and invasion of bladder cancer cells. By binding to E2F1 and Sp1, respectively, BIR2 and BIR3 domains initiate E2F1/Sp1-positive-feedback-loop-dependent transcription of miR-203, inhibiting Src protein translation, which leads to further MMP2-cleaved activation and BC (Bladder Cancer) invasion [13]. By suppressing miR-200a expression through PP2A/MAPKs/c-Jun pathways, the BIR region of XIAP reduces EGFR translation and subsequently facilitates anchorage-independent growth of BC cells [34]. The RING domain, on the one hand, contributes to AI (Anchorage-Independent) growth through stabilizing c-Myc protein by inhibiting its phosphorylation at Thr-58 [35]. On the other hand, it enhances miR-4295 transcription in a Sp1-dependent manner, which targets the 3′-UTR of p63α mRNA, thereby reducing p63α protein translation and enhancing urothelial cell transformation [36]. The two cases mentioned above link BIR3 and RING domains to transcription factor Sp1, illustrating the close relationship between Sp1 and XIAP, as well as revealing that transcription factors such as Sp1 are critical effectors of XIAP.

Additionally, RhoGDIβ was found to be a key downstream regulator of XIAP, promoting BC invasion in vivo and in vitro when XIAP was over expressed. In N-butyl-N-(4-hydroxybutyl) nitrosamine (BBN) treated mice, up-regulated XIAP stabilized nucleolin mRNA via Erks, which further increased RhoGDIβ mRNA stability, BC invasion and lung metastasis [37]. Long non-coding RNA, SNHG1, was reported to promote BC invasion and autophagy. Whereas, knocking down of SNHG1 in U5637 significantly increased the XIAP protein level, which points to a potential inhibitory role of lncRNA SNHG1 on XIAP [38].

### 3.2. Breast Cancer

XIAP was highly expressed in human breast cancer cell lines and tissue samples from patients. Foster et al. showed that the breast cancer biopsies exhibited increased XIAP in contrast to normal level in non-cancerous breast tissues [39]. Through Kaplan–Meier survival analysis and multivariate analysis, Wang et al. found that a high XIAP expression level was associated with poor prognosis [40]. This research validated that XIAP was a reliable bio-marker for cancer diagnosis and prognosis. Although the mechanisms of how XIAP was increased in cancers is not known, preliminary studies have shown it can be expressed by transcription factors such as NF-κB. In turn, high expression levels of XIAP lead to TAK1 ubiquitylation and degradation, which further blocks JNK activation, NF-κB activation stimulated by JNK and the formation of a feedback loop (Figure 3) [21]. Other transcription factor, such as RPS3 (Ribosomal Protein S3), facilitated XIAP protein expression in a NF-κB independent manner [41]. Collectively, XIAP was shown to be involved in cascades of many transcription factors. It either modulates the activation of these transcription factors, or is regulated by these factors.

Aggressive breast cancer is mainly regulated by the MNK/XIAP/NF-κB axis, MNK (MAPK iNteracting Kinase) activation increases the XIAP protein, facilitating the interaction between BIR1 domain and TAB1 (TGFβ-associated binding protein) together with its cognate kinase TAK1. This binding further contributes to the phosphorylation of NF-κB activating kinase, IKKβ, and then NF-κB is released into the nucleus activating expression downstream genes and contributing to tumor cell proliferation, growth and migration [42]. XIAP, working as a bridge, links MAPK signaling and NF-κB hyperactivation, facilitating breast cancer progression. XIAP was also reported to promote breast cancer cell proliferation, enhanced viability, and colony formation via its E3 ligase activity, leading p62 to ubiquitin-proteosome degradation (Figure 3) [43].

### 3.3. Lung Cancer

In lung cancer, micro RNAs seem to be a dominant initiator of XIAP, regulating cell proliferation and apoptosis. Several micro RNAs, such as miR-142 and miR-CHA1, were reported to participate in XIAP-mediated cell proliferation and apoptosis in human lung cancer [44,45]. Hence, RNA mimics targeting XIAP are being intensely studied for their potential utilization in cancer therapy. In addition, the XIAP/TAK1/NF-κB pathway is being studied in lung cancer as well. BMP (Bone Morphogenetic Proteins) and TGFβ specifically activate XIAP/TAK1/Id1 signaling and increase cellular resistance to cell death and drug sensitivity, however this activation undoubtedly increases carcinogenesis and provides an opportunity for activated cells to escape the toxicity of drug treatment [46]. Apart from the classic modulating pathway of caspase-3/-7/-9, caspase-8 was involved in enhancing apoptosis in human non-small cell lung cancer upon retinoic acid-inducible-gene-I-like receptor agonist and ionizing radiation cotreatment [47]. Cellular caspase-8 and caspase-9 function differently at distinct stages, the triggers they respond to may differ as well. Caspase-8 depression induced by Bcl-2 may account for drug resistance in lung cancer cells as well [48].

### 3.4. Colon Cancer

In colon cancer, up-regulation of XIAP inhibits not only apoptosis but also autophagy [49]. TGFβ is thought to increase XIAP level upstream by extracellular signals in colon cancer. Attenuation of TGFβ signaling leads to IRS-1 (Insulin Receptor Substrate-1) activation through phosphorylated Smad3, further increasing XIAP protein level and cell survival [50]. Previous studies demonstrated a critical role of the BIR domain in promoting colon cancer anchorage-independent growth and G1/S phase transition through E2F1/Cyclin E axis. When the RING domain loses its function, the BIR domain would allow XIAP to enter the nucleus and regulate E2F1 transcriptional activity via binding [51]. This mechanism may be applicable to many XIAP-RING-domain-mutations-caused diseases.

Except for XIAP’s canonical function in apoptosis and autophagy, the role XIAP plays in disrupting the success of chemotherapy has been widely studied in colon cancer. Generally, XIAP exhibits high expression, specifically in cancer cells, contributing to cancer progression by modifying cell’s resistance to death. The resistance to death renders cells with greater uncontrolled proliferation, invasion and metastasis. Most chemotherapeutic drugs kill cancer cells by cytotoxicity. Normally, cells would combat the cytotoxic stimuli by stressing themselves. When the stimuli overloads, cells would exhibit programmed cell death either by apoptosis, necroptosis or autophagy. XIAP directly amplifies the extent of cellular chemo-resistance by inhibiting the cell death pathways, which unintentionally enhances their resistance to programed cell death. Research has revealed that, HtrA1 down-regulation increases cisplatin resistance by enhancing XIAP protein stability through activating PI3K/AKT pathways [52]. Another study by Zhang et al. Demonstrated that AKT inhibitor, MK2206, reversed miR-587-conferred 5-FU resistance. miR-587 increased AKT activation through restricting PPP2R1B, thus showing that the phosphorylation of AKT was crucial for maintaining XIAP protein stability [53].

### 3.5. Other Cancers

In prostate cancer, RNA binding protein FUS, together with a circular RNA (circRNA0005276), can directly bind to the XIAP promoter region, facilitating transcription of XIAP mRNA and thus, increasing the XIAP protein level [54]. Many micro RNAs, such as miR-137, influenced the mRNA stability of XIAP by binding to its 3′-UTR in ovarian cancer [55]. It is believed that XIAP participates in raising cellular resistance to chemotherapy in ovarian cancer, since miR-519d and miR-149 sensitized ovarian cancers to cisplatin-induced cytotoxicity by suppressing XIAP [56,57]. Simultaneous inhibition of XIAP and Survivin dramatically reduced cell proliferation and increased apoptosis in pancreatic cancer cells (Panc-1), mediated by the PTEN/PI3K/AKT pathways [58]. Treatment with XIAP inhibitors brought significant reduction in malignant proliferation in a series of cancers, including gastric cancer, leukemia, and others [59,60].

## 4. XIAP: A Potential Anti-Tumor Target

XIAP, by inhibiting cell death and facilitating pro-survival pathways, promotes tumor initiation, promotion and progression. Given the detrimental nature of XIAP and mounting data linking XIAP to various types of cancers, focus has been directed to the development of anti-XIAP drugs.

### 4.1. Inhibitors or Antagonists of XIAP

Elevated XIAP protein level promotes tumor invasion and metastasis. Drugs targeting XIAP for preventing cancer progression have been identified. At present, anti-XIAP drug development mainly centers around antisense oligonucleotides and small molecule antagonists.

The antisense oligonucleotides, such as siRNA (small interfering RNA) or shRNA (short hairpin RNA), specifically bind to mRNA of XIAP. They either silence the mRNA or impair its stability, resulting in decreased XIAP protein levels. While constructing the interfering RNA is not challenging, its delivery into cells in vivo without compromising the efficiency is met with difficulty. Chemical modification and lipid particle encapsulation technologies are harnessed in modern nucleotide therapy. The former prevents nucleotides from degrading and the latter ensures efficient uptake [61]. AEG35156, a second-generation XIAP antisense oligonucleotide, is being tested for multi-purpose anti-tumor use in clinical trials [62].

Small molecule antagonists are synthetic compounds that encompass functional binding sites as substrate and can competitively bind to the target protein. Molecular antagonists of XIAP competitively occupy XIAP, and affect caspase activity. Thus far, the most promising small molecule antagonists of XIAP are SMAC mimetics. SMAC is a mitochondrial inter-membrane protein and also an endogenous XIAP inhibitor released by mitochondria in response to cell death signals. It induces cell death by binding and inactivating XIAP protein, preventing it from inhibiting apoptosis. According to 3D model analysis of SMAC protein structure and spatial conformation of a SMAC–XIAP complex, SMAC mimetics are being intensely studied for their therapeutic use to block the cell death inhibition of XIAP. They mimic the binding site of SMAC to XIAP at four amino acids, and specifically recognize and bind to the BIR2 and BIR3 domain of XIAP [16,63]. A number of SMAC mimetics have been tested clinically for dose limitation and safety, as summarized in Table 2. In addition to the pioneering human clinical trials, several preclinical studies using SMAC mimetics to switch on cell death are being conducted in animals. SMAC mimetics facilitate apoptosis not only from inactivation of XIAP but also from induction of FADD-RIP1-Caspase8 complex formation, which subsequently releases caspase-8 to produce caspase-3/7 and then induces apoptosis. Additionally, SMAC mimetics free NIKs, which subsequently lead to non-canonical NF-kB activation by facilitating cIAPs degradation [64].

### 4.2. XIAP Promotes Cellular Resistance to Cancer Therapy

XIAP contributes to the tumor development and increases the resistance of oncogenic cells to cancer treatments, among which the most extensively studied are chemotherapy and radiotherapy. High XIAP levels have been reported to be associated with chemo-therapeutic sensitivity, particularly cytarabine and other nucleosides [32]. Sraraei et al. found that several anti-leukemic agents acting via XIAP down-regulation rescued the efficacy of TRAIL (TNF-related apoptosis-inducing ligand) in leukemia [65]. Specific siRNA, targeting XIAP, promoted apoptosis and enhanced cellular sensitivity to paclitaxel in ovarian cancer environment [66]. A shift in balance between XIAP and SMAC proteins accompanied the degree of radiotherapy resistance in rectal cancer [67]. These studies demonstrated the role of XIAP in cellular resistance to chemo- and radio- therapeutics and pointed out that the combination of down-regulated XIAP and anti-tumor drugs achieved synergistic efficacy.

Prior to developing anti-XIAP agents, the mechanism of how these agents induce resistance through XIAP should be considered and understood. When studying the resistance mechanism caused by histone deacetylase (HDAC) inhibitor, JNJ-2648158 investigators found that activated c-Fos facilitated AP-1 generation and subsequently promoted XIAP transcription [68]. The clinical outcome of JNJ-2648158 was impaired by its transcription factor activating role and subsequent elevation of XIAP expression. Pretreatment with the JNK inhibitor, SP600125, rescued the cisplatin induced decrease in XIAP and other drug resistance associated genes at both the mRNA and protein levels in A549 and H446 cells [69]. This reinforced the notion that lung cancer resistance to cisplatin was mediated by the JNK-XIAP axis. The majority of XIAP function studies in tumors, concluded that XIAP is key regulator, potentiating tumor cells to survive. However, studies like these also showed that single XIAP elevations did not confer chemoresistance. Seeger et al. mocked XIAP expression level in tumor cells, they found that long-term XIAP overexpression actually did not increase resistance to chemotherapeutics. However, this kind of homeostasis could be disturbed by a XIAP antagonist such as SMAC, which means that XIAP dysregulation in tumor must be accompanied by the disturbance of its modulators [70]. Development of XIAP as a good prognostic marker, requires more intense study of XIAP and its many modulators.

### 4.3. Novel Drug Development by Targeting XIAP

It has been suggested that the advantages of inhibiting XIAP outweigh those of antagonizing any other IAPs for several reasons: (1) High abnormal expression of XIAP has been linked to poor prognosis of several types of cancers, including colon cancer, bladder cancer, breast cancer and others. [42,73]; (2) Elevated expression levels of XIAP were positively related to resistance to chemotherapy and radiotherapy [67,74]; (3) In addition, XIAP is implicated to modulate tumor cells resistance to immunotherapy, with evidence showing that XIAP is responsible for antibody-dependent cellular cytotoxicity in caspase-dependent and -independent mechanisms in inflammatory breast cancer cells [75]. These findings emphasize the detrimental effect of XIAP and highlight the importance of XIAP inhibitors or antagonists in chemotherapy.

However, elevated XIAP alone does not enhance the pro-survival rate of tumor cells to chemotherapeutic drugs, although XIAP overexpression has been identified in many tumors and is believed to be the reason for raising chemo-resistance, but chemo-resistance reversal will not occur by only down-regulating XIAP. The upstream and downstream regulators or antagonists of XIAP must be considered when developing new drugs.

The characterization of XIAP to be a drug target for cancer therapy occurred more than ten years ago [76]. Many drugs like small molecule antagonists and antisense oligonucleotides have moved into phase II or III clinical trials, as listed in Table 2. Next-generation novel drug development adopts fragment-based and structure-based methods incorporating X-ray crystallography, computational analysis and NMR solution conformational studies. Potent non peptidomimetic small molecule orally bioavailable antagonist inhibiting both XIAP and cIAP1 have been identified [77]. Many of the known anti-XIAP drugs have more than one target, for instance ASTX660 can antagonize both XIAP and cIAP1/2 [78]. Will drugs with multiple IAP targets achieve a better clinical effect than those that only target XIAP? Another consideration is what part of the XIAP molecule should be targeted. The caspase inhibition is mainly carried on by BIR2/3 domains, the linker upstream of BIR2 domain is believed to be the most essential site when interacting with caspase-3/7. Should the new drug occupy all the possible caspase binding sites to achieve the best inhibition efficacy? Finally, as a compact protein, XIAP has its own way of undergoing conformational changes when interacting with other molecules, but very little is known about this process.

## 5. XIAP and Non-Coding RNA

XIAP has been shown to interact with various proteins, such as caspases, SMAC, etc. In recent years, our understanding of how XIAP interacts with these different molecules has improved considerably. In addition to protein–XIAP interactions, it has been found to interact with RNAs. Among these XIAP-binding RNAs, the most interesting ones are those involving non-coding RNAs because they bring new insight into how XIAP is regulated in cells.

The most common RNAs are messenger RNA (mRNA), transfer RNA (tRNA), and ribosomal RNA (rRNA). XIAP has been found to interact with non-coding RNAs which can be divided into two subtypes: structural non-coding RNA and regulatory non-coding RNA. Among the regulatory non-coding RNA, numerous small non-coding RNAs and long non-coding RNAs have been found to impact gene expression during transcription and translation. The regulatory non-coding RNAs are also involved in XIAP regulation during cancer progression. They either influence the integrity of XIAP mRNA or act as effectors of XIAP to amplify signals through binding to their 3′-UTR. Although non-coding RNAs do not directly translate into proteins, they work actively in distinct ways that impact cellular function.

### 5.1. Long Non-Coding RNA

Non-coding RNA with a length over 200nt are classified as long non-coding RNA (lncRNA). lncRNA account for the majority of the ncRNAs and are essential for regulating gene expression [79]. Emerging evidence demonstrates that lncRNA may be a key link to tumorigenesis, and XIAP is one of the proteins they target.

Long non-coding RNA CRNDE (Colorectal Neoplasia Differentially Expressed) was the first lncRNA reported to regulate XIAP. CRNDE was found to decrease the XIAP protein level by negatively regulating miR-186 which specifically binds to the 3′-UTR of XIAP and impairs its stability in glioma stem cells [80]. Sp1 is a well-known transcription factor that can specifically bind to the XIAP promoter region and facilitate XIAP gene transcription. lncRNA XIAP-AS1 was found to interact with Sp1 and thereby improve XIAP transcription level and reduce apoptosis in gastric cancer cells [81]. Recently, XIAP-AS1 has been found to play an oncogenic role by interfering with STAT3 (Signal Transducer and Activator of Transcription 3) phosphorylation and EMT (Epithelial–Mesenchymal Transition) markers expression [82].

### 5.2. Micro RNA

Ectopic expression of XIAP 3 prime untranslated region improves cell proliferation, tumor growth, colony formation, migration and invasion in human breast cancer. The 3′-UTR of XIAP itself functions as ceRNA (competing endogenous RNA) and thus, frees FSCN1 from miR-29a-5p, which increases the motility of cells in breast cancer [83]. miR-200 is a set of micro RNA family involved in cancer metastasis. This family includes: miR-200a, miR-200b, miR-429, miR-200c, miR-141. Among them, miR-200a and miR-200c were reported to participate in XIAP cascades in bladder cancer cells. In particular, miR-200c directly binds to 3′-UTR of XIAP and reduces XIAP mRNA stability. Thus, down-regulation of miR-200c stabilizes XIAP mRNA and facilitates BC invasion and lung metastasis [84]. The number of micro RNA that have been reported to have direct interaction with XIAP is increasing.

### 5.3. Circular RNA

As single stranded polynucleotides, secondary structures of RNA enable them to pair with themselves, rendering the RNA with enhanced stability. Except for most linear RNA, another type of RNA that forms a covalently closed loop with its 3′- and 5′- ends adhered together is called circular RNA (circRNA). Most types of circRNAs are classified as non-coding RNA and regulate gene expression. Although, some of them have been shown to encode proteins, gene expression regulation remains their prominent role [85].

Zhang et al. first revealed that over-expression of Circ_0005015 led to increased XIAP expression by inhibiting miR-519d-3p activity [86]. The positive correlation of circRNA0005276 and XIAP was mediated by FUS. circRNA0005276 promoted XIAP transcription and facilitated proliferation and migration of prostate cancer cells [54]. By targeting XIAP, circPAN3 enhanced chemo-resistance in acute myeloid leukemia with miR-153-3p/miR-183-5p as intermediates [87]. Very limited studies have been carried on the relationship between circular RNA and XIAP. No such evidence has been found in support of circular RNA’s ability to directly couple with XIAP. Micro RNAs tend to be the dominating intermediate that regulate XIAP.

## 6. Conclusions and Discussion

Originally defined as apoptosis inhibitor protein, XIAP fulfills its mission by binding with apoptosis initiator caspase-9 and effector caspase-3/7, marking them for proteosome degradation by ubiquitination. However, as research on XIAP progresses, XIAP has been found to be involved in many other cell death pathways, including autophagy, necroptosis and copper homeostasis. When it comes to XIAP, it should not be regarded only as apoptosis inhibitor, but rather as a master cell death resistance regulator.

Due to its critical position in preventing cell death, over-expression of XIAP enhances cell tolerance to external and internal stimuli. XIAP over-expression has been validated in numerous types of cancer cells with qRT-PCR and Western blot, and has been linked to malignant transformation, increased proliferation and metastasis. Studies on how XIAP is up-regulated include transcription factors, TNF/JNK/NF-κB signaling pathways, together with various non-coding RNA. More and more studies rendered XIAP a reliable bio-marker for cancer diagnosis and prognosis.

A number of drugs targeting XIAP are under development, which are mainly antisense oligonucleotides and small molecule antagonists. Several of them have been shown to be effective clinically. Combined administration of cancer treatments together with anti-XIAP drugs are being tested. Differentiated XIAP expression profile among cancer patients assigned the higher XIAP expression subgroup for worse outcome and poor prognosis. XIAP is a key target for therapy and also an essential factor for promoting resistance to cancer treatment. It is well supported that XIAP can make cells less sensitive to chemotherapy, radiotherapy and immunotherapy. Anti-tumor agents unavoidably trigger an XIAP induction cascade, either by activating XIAP transcription factors or by other molecular signaling pathways that can upregulate XIAP. Participation of XIAP during cancer development makes the situation more complex for remedy and reduces the overall survival rate.

Apart from canonical protein regulatory pathways that target XIAP, many non-coding RNAs are occupying an increasingly significant position in XIAP regulation. Non-coding RNA revealed new mechanisms of XIAP regulation, with micro RNA acting on messenger RNA stability by binding to the 3′-UTR of XIAP mRNA. Since the circRNA’s role and lncRNA’s role in mediating XIAP pathways still remain elusive, more work should be carried out in this area.

## Figures and Tables

**Table 1:** The binding substrates of each domain in XIAP.

Domain	Binding Substrates	Reference
BIR1	TAB1, BIR1	[10,11]
linker-BIR2	Caspase-3/7, E2F1, HAX-1, SMAC	[12,18,19,20]
BIR3	Caspase-9, Sp1, ARTS, HAX-1, SMAC, BIR3	[18,19,20,21,22]
UBA	Ubiquitin, UBA	[9]
RING	Multiple protein substrates and plays E3 ligase activity	[10,23]

**Table 2:** The research progression on XIAP-related drugs. Data are summarized according to U.S. National Library of Medicine (https://clinicaltrials.gov/ct2/home) in September 2020.

Drug	Stage	Group and Reference
AEG35156/GEM640	Phase1–2 complicated	Aegera Therapeutics, Inc.
ASTX660	Phase 1–2 recruiting	Astex Pharmaceuticals, Inc.
ASTX727	Phase 2	Astex Pharmaceuticals, Inc.
AT-406/Debio 1143	Phase 1 complicated	Debiopharm International SA.
LCL-161	Phase 2 complicated	Novartis Pharmaceuticals.
GDC-0152	Phase 1 terminated	Genentech, Inc.
TL32711/Birinipant	Phase 1–2 complicated	TetraLogic Pharmaceuticals.
HGS1029	Phase 1 complicated	Human Genome Sciences Inc.
SM-164	Preclinic	[71,72]
